# Effects of indoor plants on CO_2_ concentration, indoor air temperature and relative humidity in office buildings

**DOI:** 10.1371/journal.pone.0305956

**Published:** 2024-07-17

**Authors:** Junzhiwei Jiang, Peter Irga, Robert Coe, Philip Gibbons

**Affiliations:** 1 Fenner School of Environment and Society, Australian National University, Canberra, ACT, Australia; 2 Faculty of Engineering and Information Technology, Plants and Environmental Quality Research Group, School of Civil and Environmental Engineering, University of Technology Sydney, Sydney, NSW, Australia; 3 Stacked Farm, Arundel, Gold Coast, Queensland, Australia; Satyawati College, University of Delhi, INDIA

## Abstract

This experimental study investigates the influence of indoor plants on three aspects of air quality in office spaces: relative humidity, indoor air temperature, and carbon dioxide concentration. Employing a Latin square design, we rotated three different treatments across three offices over six time periods. These treatments included a control (no plants), a low-volume treatment (five plants), and a high-volume treatment (eighteen plants) of *Nephrolepis exaltata* (Boston fern). Air quality parameters were continuously monitored at five-minute intervals using Trace Gas Analyzers. Generalised linear mixed modelling (GLMM) was employed to examine the effect of each treatment on relative humidity, indoor air temperature and CO_2_ concentration. We observed a significant positive correlation between the number of indoor plants and relative humidity levels. In offices without any plants, the median relative humidity was 29.1%. This increased to 38.9% in offices with 5 plants and further to 49.2% in offices with 18 plants. However, we did not find significant associations between the number of indoor plants and indoor air temperature or corrected CO_2_ concentration. Our research provides support for the use of indoor plants to increase relative humidity, which can have health benefits in dry climates, but does not provide support for using indoor plants to regulate indoor air temperatures or CO_2_ concentration in office environments.

## Introduction

Indoor air quality is a critical issue in the workplace because it is where individuals spend a significant amount of time [1]. Poor indoor air quality can lead to a range of health problems, including respiratory illness [2], headaches [3] and fatigue [4]. This, in turn, can harm productivity, job satisfaction and the overall well-being of employees [5,6]. Indoor plants have been suggested as an alternative or additional approach to mechanical ventilation systems to address these issues [7]. Indoor plants are affordable and can enhance the aesthetics of indoor spaces. Besides possibly improving air quality, they can also boost people’s well-being at work [8,9] and offer a more sustainable alternative to traditional air conditioning systems [10]. Three aspects of indoor air quality that can potentially be improved with plants are carbon dioxide (CO_2_), indoor air temperature and relative humidity.

Elevated levels of carbon dioxide within indoor environments can have a number of negative effects on health and productivity. Most countries have established regulatory standards and guidelines aimed at maintaining carbon dioxide concentrations below a specified threshold (typically around 850 to 1500 ppm) in buildings such as offices, schools, and residences [11–13]. Air exchange through ventilation may not be sufficient alone to keep carbon dioxide within these thresholds [14,15]. For example, Wu et al.[16] recorded levels of carbon dioxide concentration in offices of ordinary buildings (1294 ppm) that exceeded the standard set for office environments in China by 7.2%. Long exposure to elevated levels of carbon dioxide can significantly reduce human vigilance [17]. Jin et al. [18] suggested that 40 minutes of exposure to 4000 ppm CO_2_ concentration can affect daytime alertness and neural activity. Maniscalco et al. [19] found that exposure to high levels of CO_2_ can significantly affect cognitive performance, with participants showing decreased performance on tasks related to attention, memory, and decision-making. Plants absorb carbon dioxide through photosynthesis and release oxygen, thereby effectively reducing the concentration of carbon dioxide in the surrounding environment [20]. There are several publications that discuss how a single or small number of plants can effectively decrease the concentration of CO_2_ in experimental chambers [21–24], but the effect of indoor plants on CO_2_ has not been widely tested within office environments [25,26]. However, Tudiwer et al. [26] found that the CO_2_ concentration of classrooms were reduced by an indoor living wall system compared to non-greened classrooms, even when the volume of the plants was only around 1% of the room’s volume.

Relative humidity and temperature can also affect indoor air quality. Low humidity levels have been associated with a range of health effects in working spaces, including dry mucous membranes, colds, eye complaints, and skin complaints [27]. High air humidity levels combined with high indoor air temperatures can cause discomfort, exhaustion, and can exacerbate allergies and respiratory diseases [28]. Plants have the ability to regulate indoor relative humidity and indoor air temperature through the process of transpiration and evaporation of soil moisture [29]. Fernández-Cañero et al. provided evidence of this phenomenon, indicating that the incorporation of plants can engender a reduction in average room temperatures by 4°C alongside an augmentation in overall humidity [30]. Su and Lin [31] demonstrated that green walls could lower indoor temperatures by 2°C and enhance relative humidity by 10%. Smith et al. utilized indoor plants to elevate relative humidity from levels below 40%, effectively bringing it into the recommended range of 40–60% [32]. Kim et al. also reported that when *Epipremnum aureum* (Golden Pothos), *Rosmarinus officinalis* (Rosemary), and *Gardenia jasminoides* (Cape Jasmine) occupied 3%, 6%, and 9% of indoor spaces, they respectively increased humidity by 4.8%, 8.3%, and 10% [33].

Although there is some evidence that indoor plants can improve these three aspects of air quality (i.e., carbon dioxide, indoor air temperature and relative humidity), there are few experimental studies have been conducted to determine their impact on indoor air quality in real-life settings such as offices. Lohr and Pearson-Mims [34] observed that the presence of indoor plants, accounting for 5% of the office volume, led to higher humidity levels. Husti et al. [35] reported notable carbon dioxide removal rates of 58.33% in offices with *Ficus elastica*, *Dracaena deremensis*, and *Sansevieria trifasciata*. A study by Han et al. [36] compared a room with three pots of *R*. *hainanensis* Merr. versus one pot, it was found that the former resulted in significantly higher CO_2_ concentration and humidity. However, some studies could not detect positive effects of plants on different aspects of indoor air quality [23,26,37,61] and thus a consensus has yet to emerge on the effect of indoor plants on indoor air quality.

To add to this body of evidence, we established a controlled experiment within working offices to investigate the effects of indoor plants (specifically *Nephrolepis exaltata*) on relative humidity, indoor air temperature and carbon dioxide in office environments.

## Materials and methods

### Study area

The study was conducted in three offices located within a six-green-star-rated building in Canberra, Australia (35.2809° S, 149.1200° E). Canberra is characterized by a mean minimum temperature of 7.1°C, a mean maximum temperature of 20.0°C, a mean minimum outdoor relative humidity of 48.5% and a mean maximum outdoor relative humidity of 72.5% [38].

We kept the conditions of the three offices as similar as possible throughout the experiment. The volume of the three offices ranged from 28.8 to 33.4 m^3^, they had the same ceiling height (3.0 m) and window area (4.3 m^2^), and all faced north, thus receiving approximately the same amount and intensity of natural light throughout the day. To prevent confounding factors related to light and CO_2_ levels, we kept the lights turned off, the air conditioning turned off, the door closed, and no people were permitted to enter for each measurement period.

### Experimental design

We designed the experiment using a Latin square design. As detailed in Table 1, each of the three office spaces was randomly assigned one of the three treatments in each experimental period. In the Latin rectangle design, treatments are randomly assigned to columns (offices) and rows (periods) (Table 1), thereby effectively controlling for confounding variables (in this case the effect of the different offices and different periods of the experiment). We replicated the Latin square twice. We further accounted for possible confounding between different offices and periods by taking baseline measurements of air quality in each office on the day prior to installing the treatments and compared this with measurements taken in the same office on the day when the treatments were installed.

**Table 1:** Treatment allocation within the Latin rectangle design (A = control, B = 5 plants, C = 18 plants).

	office I	office II	office III
Period 1	A	B	C
Period 2	B	C	A
Period 3	C	A	B
Period 4	A	B	C
Period 5	B	C	A
Period 6	C	A	B

Key: A = Control (zero plants); B = Low volume of plants (five plants); C = High volume of plants (eighteen plants).

The three treatments involved varying quantities of *Nephrolepis exaltata* (Boston fern or sword fern) added to offices. This evergreen plant is renowned for its adaptability to indoor environments and indoor decoration. Its widespread use worldwide attests to its economic feasibility and accessibility, making it a viable option for potential large-scale office implementation. The rationale for employing *Nephrolepis exaltata* for this study was also informed by previous research, which highlighted its effectiveness in air purification within a chamber setting [31]. The experiment included a control treatment with no plants, a low-volume treatment with five pots of plants, and a high-volume treatment with eighteen pots of plants. The mean weight of each pot (plant, soil and pot) was 3154 grams. The interior Green View Index [39], the ratio of plant pixels to the total pixels in a 360° panoramic image of the office, was 3.45 ± 1.04% for the low-volume treatment, and 11.82 ± 1.47% for the high-volume treatment. The leaf area on average was 2.247 ± 1.09 cm^2^, which was measured using ImageJ analysis software (version 1.54f) [40]. The same volume of water was added to each pot twice a week.

The experiment was conducted on two consecutive days over six periods from August 20 to September 25, 2022. In each of the six periods of the experiment, indoor air temperature, relative humidity and CO_2_ concentration were monitored from 09:00 to 17:30 in each office the day prior to installing the treatment and from 09:00 to 17:30 the following day after each treatment was installed.

### Measured variables

Relative humidity, indoor air temperature and CO_2_ concentration were monitored in each office at five-minute intervals using Trace Gas Analyzers LI-6400/XT (LI-COR, USA). The analyzer measures relative humidity (%) with an accuracy of ± 2%; CO_2_ concentration (ppm) with an accuracy of ± 50 ppm; and indoor air temperature (°C) with an accuracy ± 0.5°C. The devices were deployed at 0.8 m in height in the middle of each office, which is the approximate respiratory zone of an individual seated in an office chair. We applied corrected CO_2_ concentration data to estimate the CO_2_ level indoors. The need for this correction stems from disparities between open-path and closed-path measurement techniques. As identified by Burba et al. [41], substantial discrepancies can emerge when the instrument surface temperature deviates significantly from the ambient temperature. This can cause variations in sensible heat fluxes inside the open-path cell, as compared to the ambient air. Such variations can, in turn, influence the CO_2_ concentrations detected.

We summarized each air quality parameter as its minimum, median, maximum and interquartile range (IQR) based on the values collected every 5 minutes over the course of each day. The pre-experiment day and experimental day for each indoor air quality parameter were then calculated and used for subsequent statistical analyses. Additional variables recorded were the experiment date (Period1, Period 2,… Period 6), the specific treatment (control, low volume, or high volume of plants), the experiment phase (pre-experiment day or experimental day), and the office number (Office I, Office II, or Office III). These were used as independent variables in the subsequent statistical analyses.

### Statistical analysis

We fitted generalized linear mixed models (GLMMs) to identify the effect of adding different numbers of plants to offices on minimum, median, maximum, and IQR for each of relative humidity, indoor air temperature, and CO_2_ concentration. We employed multi-model inference to identify whether there was a treatment effect for each of the measures of air quality. We compared four candidate models (Table 2): (1) a null model including only ’office’ and period’ which were fitted as random effects because of multiple observations within each office and period respectively; (2) a model in which ‘treatment’ was added as a fixed effect; (3) a model in which the timing of the measurement relative to when the treatment was introduced (i.e., ‘before’ or ‘after’) was also added; and (4) a model that tested an interaction between ’treatment’ and the timing of the measurement (i.e., ‘before’ or ‘after’), to examine if the measures of air quality also varied before and after the treatments were introduced to the offices. When this last model has empirical support, it indicates there is a treatment effect for the air quality measure. Models were ranked by comparing their Akaike Information Criterion corrected for small samples (AICc) and derived AICc differences. Models within 2 AICc points of the highest-ranked model were considered to have empirical support. All analyses were conducted in R [42] using the glmmTMB [43], MuMIn [44], ggplot2 [45] and ggeffects [46] packages.

**Table 2:** Generalized linear mixed models of interior greenery effect. A “+” symbol indicates the variable was included in the model.

Model	Random Effects		Fixed Effects		Interaction
	Office	Period	Treatment	Before_After	Treatment * Before_After
glmm 1	+	+			
glmm 2	+	+	+		
glmm 3	+	+	+	+	
glmm 4	+	+	+	+	+

## Results

### Relative humidity

The minimum, median, maximum and IQR of relative humidity averaged across all offices and days of the experiment are summarized in Table 3. The relative humidity did not reach above 70% during the entire measurement period.

**Table 3:** Results of relative humidity of the three treatments. Each value is averaged across the three offices and six separate days the treatments were installed.

Number of plants		Pre-experiment	No plants	5 plants	18 plants
Relative humidity (%)	Min	20.0	23.6	25.4	27.4
	Median	29.3	29.1	38.9	49.2
	Max	43.6	35.0	50.8	57.5
	IQR	8.3	3.8	10.4	18.8

**Fig 1:**
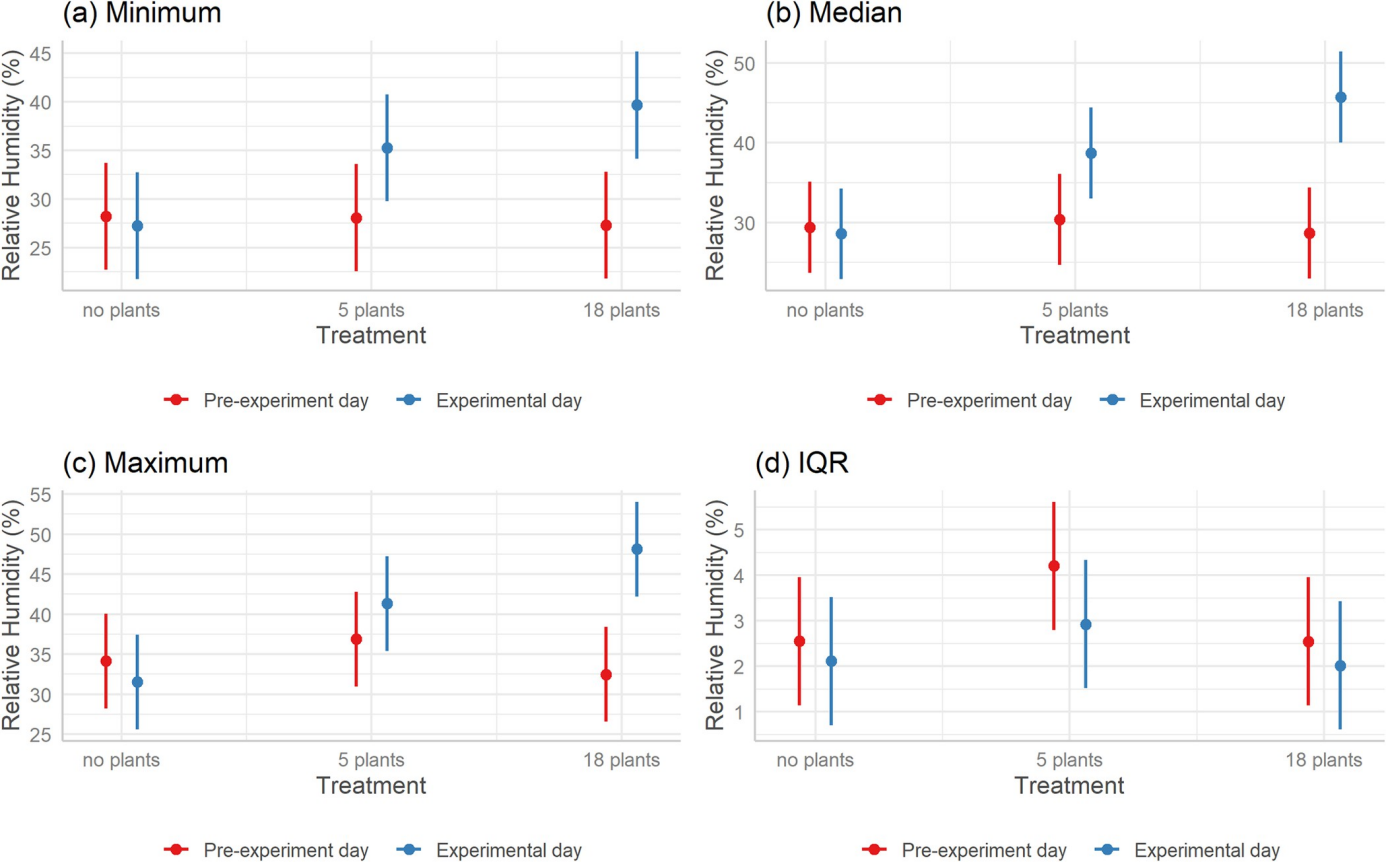
Predicted (a) minimum, (b) median, (c) maximum, and (d) IQR of relative humidity across different treatments using the highest-ranked GLMM for each variable. Red bars represent the pre-experiment day and blue bars represent the experimental day the treatment was installed. Error bars are 95% confidence intervals.

The only model with empirical support (AICc difference ≤2) for minimum, median, and maximum humidity in offices was glmm4 in Table 2, which is the model that included an interaction between treatment and if the measurement was taken before or after the treatment was introduced (Table 2). This model had an AIC Weight of 0.997, indicating there was a 99.7% chance this was the strongest model selected. Predictions from this model indicated that minimum, median and maximum humidity were higher in offices where plants were introduced relative to offices where plants were not introduced, with the highest values where 18 plants were introduced (Fig 1).

### Indoor air temperature

The minimum, median, maximum and IQR of air temperature averaged across all offices and days of the experiment are summarized in Table 4.

**Table 4:** Results of indoor air temperature of the three treatments. The treatments are 0 plants (control), 5 plants (low volume) and 18 plants (high volume).

Number of plants		Pre-experiment	0 plants	5 plants	18 plants
Indoor air temperature (°C)	Min	19.9	19.1	20.4	19.1
	Median	24.4	23.8	23.4	23.1
	Max	32.6	29.5	31.8	31.9
	IQR	4.0	4.8	4.1	4.2

**Fig 2:**
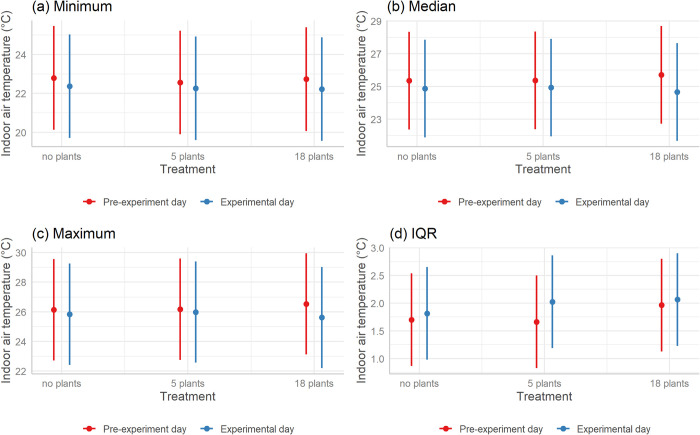
Comparison of (a) minimum, (b) median, (c) maximum, and (d) IQR of indoor air temperature across different treatments; Red bars represent the pre-experiment day; Blue bars represent the experimental day the treatment was installed. The treatments are no plants (control), 5 plants (low volume) and 18 plants (high volume). Error bars are 95% confidence intervals.

The null model (AIC Weight 0.846) in Table 2 (glmm1) was the top-ranked model and none of the other models had empirical support (AIC differences >2) indicating that introducing plants did not significantly affect the minimum, median, maximum, or IQR of indoor air temperature (Fig 2).

### Corrected carbon dioxide concentration

**Fig 3:**
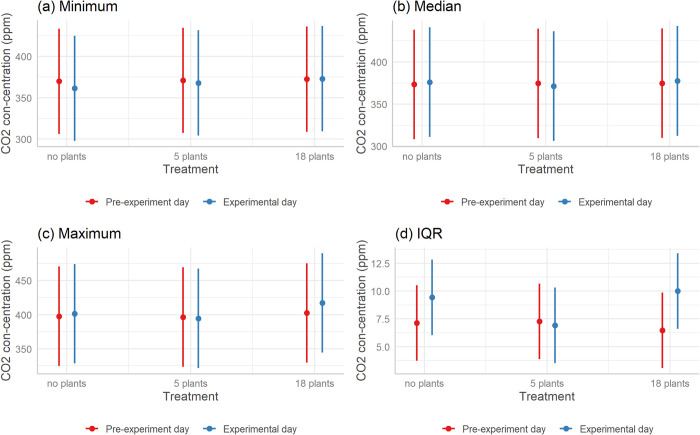
Comparison of (a) minimum, (b) median, (c) maximum, and (d) IQR of corrected CO_2_ concentration across different treatments. Red bars represent the pre-experiment day; blue bars represent the experimental day the treatment was installed. The treatments are no plants (control), 5 plants (low volume), and 18 plants (high volume). Error bars are 95% confidence intervals.

The minimum, median, maximum and IQR of corrected CO_2_ concentration averaged across all offices and days of the experiment are summarized in Table 5. The best model predicting corrected CO_2_ concentration in offices was the null model with AIC Weight of 0.898. None of the other models had empirical support (AICc difference >2). The minimum, median, maximum, and IQR of corrected CO_2_ concentration remained comparable across different office settings in offices where plants were and were not introduced (Fig 3).

**Table 5:** Results of corrected CO_2_ concentrations of the three treatments.

Number of plants		Pre-experiment	0 plants	5 plants	18 plants
CO_2_ concentration (ppm)	Min	278.52	282.91	290.14	286.96
	Median	405.18	401.74	398.03	397.40
	Max	491.70	479.23	444.64	541.51
	IQR	118.34	111.13	108.70	131.25

## Discussion

The aim of this study was to investigate the impact of indoor plants on office air quality, specifically relative humidity, indoor air temperature, and CO_2_ concentration.

The introduction of indoor plants significantly altered relative humidity in offices. Offices with more plants exhibited higher minimum, median, and maximum relative humidity values compared to those without plants (Fig 2). This could be attributed to plant transpiration, a process wherein plants release water vapour into the surrounding environment, thereby increasing ambient humidity levels [47,48]. This observation aligns with previous research that has found indoor plants can increase relative humidity, especially in dry climates [49] such as our study area. The median relative humidity in offices without plants was 29.1% (Table 3). This is lower than the recommended standard of 40%-70% [50]. Low-humidity environments can lead to respiratory health risks and skin irritation due to the drying of mucous membranes, especially for individuals spending prolonged hours in office settings [27,51,52]. Furthermore, the stability and transmission efficiency of viruses, such as the flu, are enhanced in these conditions, raising the risk of infections [53,54]. Low humidity can also impact overall comfort and well-being, affecting mood, and productivity, and leading to increased absenteeism [55–57]. In offices, low humidity poses a risk to electronic equipment through increased static electricity [58]. The median relative humidity in offices increased to 38.9% and 49.2% in offices with 5 and 18 plants respectively, indicating that the addition of a sufficient number of plants can play a vital role in elevating the relative humidity in office environments within the recommended range, mitigating these risks and promoting a healthier, more comfortable workplace. In line with our observations on the role of indoor plants in elevating relative humidity, similar findings have been reported by Park et al. [59], who found that the monthly mean relative humidity in rooms with plants was 4% higher than control rooms in one school and 8% higher than control rooms in another school. These results are also in accord with the previous work reported by Fernández-Cañero et al. [30], which highlighted an average 15% increase in relative humidity levels near indoor plants.

The influence of indoor plants on ambient temperature has been a topic of debate. While we observed that the presence of indoor plants did not significantly alter indoor air temperature, some past studies have suggested that indoor plants decrease indoor air temperatures, particularly in the context of living or plant walls [30,49,59]. However, other research suggests the opposite. Meng et al. (2022) found that the mean air temperature in rooms with a living wall was marginally higher than that in rooms without a living wall [60]. Our result that the presence of indoor plants does not induce substantial changes in indoor air temperatures was consistent with the findings of Ghazalli et al. [61] and Tudiwer and Korjenic [26], who also found that living walls did not lead to noticeable changes in indoor air temperature. The discrepancy between our findings and those of certain past studies points towards the complexity of these phenomena and suggests that various factors (e.g., ambient temperature, type of plants, positioning of plants, plant volume) may influence the effect of indoor plants on air temperature.

The third air quality parameter, CO_2_ concentration, also did not change significantly with the introduction of indoor plants in our study. Despite expectations that plants could reduce CO_2_ levels through photosynthesis, our results indicated little change in minimum, maximum or the IQR of CO_2_ concentrations in offices with plants compared to those without plants. Our findings are in contrast with several studies asserting the potential of indoor plants to lower CO_2_ concentrations [49,62]. Nonetheless, our results are in alignment with some studies that have reported minor effects of plants on CO_2_ removal in office spaces [23,37]. While plants do participate in CO_2_ absorption, the efficacy of their absorption capabilities in indoor settings is influenced by several factors including plant species, ventilation patterns, and the baseline CO_2_ concentrations present within the office milieu. For example, research has shown that certain plant species used indoors like *Nephrolepis exaltata*(Boston fern) perform less effectively in reducing CO_2_ concentration and particulate matter compared to *Epipremnum aureum* (golden pothos), *Chlorophytum comosum* (spider plant) or a combination of different plants [63]. The prominence of light conditions as the principal variable cannot be understated in the context of CO_2_ mitigation through indoor vegetation, especially given that plants can yield elevated CO_2_ during their respiration [64].

Although the impact of indoor plants on CO_2_ concentration and indoor air temperature was not significant in this study, we acknowledge the additional psychological benefits of indoor plants. There is a growing body of research that suggests the presence of plants in the office space can enhance mental well-being, reduce stress, and improve concentration and productivity [65–67]. The incorporation of plants within offices could thus serve dual purposes; not only potentially aiding in maintaining relative humidity but also in fostering a more conducive and healthier workspace for employees. Employers and facility managers might consider these findings while designing office spaces, as incorporating plants could be a cost-effective measure to contribute positively to employee health and well-being.

Our findings indicate that while 5–18 indoor plants can significantly improve relative humidity in office spaces, their effect on indoor air temperature and CO_2_ concentration was minimal, at least in the context of our study. It’s worth mentioning that the specific characteristics of the office environment, including the ventilation system, occupancy levels, and external climate conditions, might also contribute to these observations. For example, our results could be influenced by the fact that the study was conducted in a 6-star green building [68,69], which is designed with high-efficiency systems to maintain consistent indoor environmental conditions. Such buildings utilize advanced insulation, strategic window placement, energy-efficient systems, and possibly automated temperature control systems that respond to both internal and external environmental changes [70]. Given the efficiency of these systems, the minor temperature effects from the indoor plants might have been quickly counterbalanced, leading to a stable overall indoor air temperature.

This study had several limitations that should be acknowledged. First, the experiment was conducted in a limited number of office spaces over a relatively short period. The impacts of indoor plants on air quality parameters might manifest differently over an extended period or in different spaces, considering factors such as occupancy levels, usage patterns, and inherent variations in the indoor environment. Future studies could benefit from exploring a broader range of office settings over extended periods to capture more comprehensive data, including the potential seasonal variations in plant effectiveness, however, this would also introduce additional variation into the study which creates another set of challenges when executing a balanced experiment. Second, only one plant species, *Nephrolepis exaltata* (Boston fern), was used in the experiment. Different plant species have different metabolic rates, which could lead to variations in their impacts on indoor air quality parameters [21,71]. The selection of a single species also overlooks the potential synergistic effects that a variety of plants might have on indoor air quality. Future research should consider including multiple plant species to understand the diverse impacts of various indoor plants and to identify optimal plant combinations for improving air quality. Third, the study was conducted with the air conditioning units off to reduce confounding. However, many office spaces are typically air-conditioned, which can significantly influence indoor air quality parameters. This lack of typical air conditioning could limit the applicability of our findings in real-world settings. Lastly, the study was conducted only during the daytime hours, aligning with the plant’s metabolic activity. However, offices are often used beyond these hours, and hence the full impact of the plants on the indoor environment may not have been captured. Future research could address these limitations by including a wider variety of office spaces, different plant species, and varying office hours. Additionally, while this study focused on CO_2_ concentration, relative humidity, and indoor air temperature, other air quality parameters such as volatile organic compounds (VOCs) and particulate matter were not measured. These factors also play a critical role in determining indoor air quality and could be affected by the presence of indoor plants. Future studies could expand their scope to include these additional parameters for a more comprehensive assessment of indoor air quality.

## Conclusion

We examined the impact of indoor plants on air quality parameters in office environments, focusing on relative humidity, indoor air temperature, and CO_2_ concentration. Our findings demonstrated that, while indoor plants significantly increase relative humidity, they had a negligible effect on indoor air temperature and CO_2_ concentration in the offices we sampled. This suggests that while plants can enhance comfort in dry indoor environments, their role in regulating temperature and CO_2_ levels is limited. Conducted in a 6-green-star-rated building, the study’s context may have influenced these outcomes, particularly the minimal impact on air temperature and CO_2_ concentration. This highlights the need for further research in diverse settings and with different plant species to fully understand the capabilities and limitations of indoor plants in office environments. Despite the modest environmental impacts observed, the psychological benefits of indoor plants, such as improved well-being and productivity, remain a compelling argument for their inclusion in office spaces.
